# Third molar surgery: the patient's and the clinician's perspective

**DOI:** 10.1186/1755-7682-2-32

**Published:** 2009-10-24

**Authors:** Waseem Jerjes, Tahwinder Upile, Panagiotis Kafas, Syedda Abbas, Jubli Rob, Eileen McCarthy, Peter McCarthy, Colin Hopper

**Affiliations:** 1 Unit of Oral and Maxillofacial Surgery, UCL Eastman Dental Institute, London, UK; 2 UCLH Head and Neck Centre, London, UK; 3 Department of Surgery, University College London Medical School, London, UK; 4 Department of Oral Surgery and Radiology, School of Dentistry, Aristotle University, Thessalonica, Greece; 5 Department of Medicine, University College London Medical School, London, UK; 6 Faculty of Health, Sport and Science, University of Glamorgan, Pontypridd, UK

## Abstract

**Background:**

In this report, the problems of third molar surgery have been reviewed from the perspective of both patient and clinician; additionally an overall analysis of preoperative imaging investigations was carried out.

Specifically, three main areas of interest were investigated: the prediction of surgical difficulty and potential complications; the assessment of stress and anxiety and finally the assessment of postoperative complications and the surgeon's experience.

**Findings:**

In the first study, the prediction of surgical difficulty and potential injury to the inferior alveolar nerve was assessed. This was achieved by examining the patient's orthopantomograms and by using the Pederson Difficulty Index (PDI). Several radiological signs were identified and a classification tree was created to help predict the incidence of such event.

In the second study, a prospective assessment addressing the patient's stress and anxiety pre-, intra- and postoperatively was employed. Midazolam was the active drug used against placebo. Objective and subjective parameters were assessed, including measuring the cortisol level in saliva. Midazolam was found to significantly reduce anxiety levels and salivary cortisol was identified as an accurate anxiety marker.

In the third study, postoperative complications and the surgeon's experience were examined. Few patients in this study suffered permanent nerve dysfunction. Junior surgeons reported a higher complication rate particularly in trismus, alveolar osteitis, infection and paraesthesia over the distributions of the inferior alveolar and lingual nerves. In apparent contrast, senior surgeons reported higher incidence of postoperative bleeding.

**Discussion:**

These studies if well employed can lead to favourable alteration in patient management and might have a positive impact on future healthcare service.

## Findings

The removal of third molars is the most common surgical procedure practiced in oral & maxillofacial surgery (OMFS). The procedure can be implemented under anaesthesia (either local or general) or intravenous sedation.

The patient's journey starts when they present with pain, recurrent swelling or recurrent infection in the third molar area. The patient is usually referred for assessment and at this stage a decision for the removal of the third molar(s) is formulated. This is followed by three phases: the preoperative phase, the intraoperative phase and the postoperative phase.

**Figure 1:**
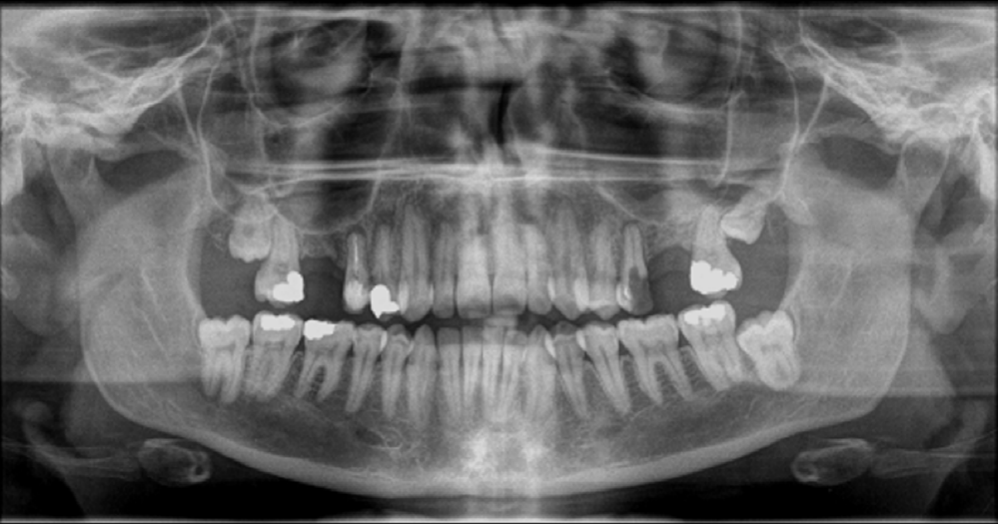
**Standard orthopantomogram (OPG)**. It is usually acquired when a patient presents with symptoms related to upper or lower jaw or near by structures.

In this report, three clinical studies were employed in an attempt to assess and hopefully lead to improvement in the patient's experience through third molar surgery. The first study was conducted in a retrospective fashion where 1934 orthopantomograms (OPGs) were examined to assess the surgical difficulty as well as the relationship between the third molar and the inferior alveolar canal (IAC), (Figure 1); those findings were then compared to the incidence of temporary and permanent inferior alveolar nerve (IAN) paraesthesia in the postoperative phase [1]. The second study was conducted in a double-blind randomized controlled fashion to assess the patient's stress and anxiety pre-, intra- and postoperatively. Several objective and subjective parameters were used to assess stress and anxiety; including the use of salivary cortisol markers. In the same trial, the efficacy of pre-emptive sublingual midazolam was assessed against placebo [2]. The third study prospectively assessed the postoperative complications in 1087 patients [3]; including the surgeon's experience parameter and its potential effect on the complications rate [4].

**Figure 2:**
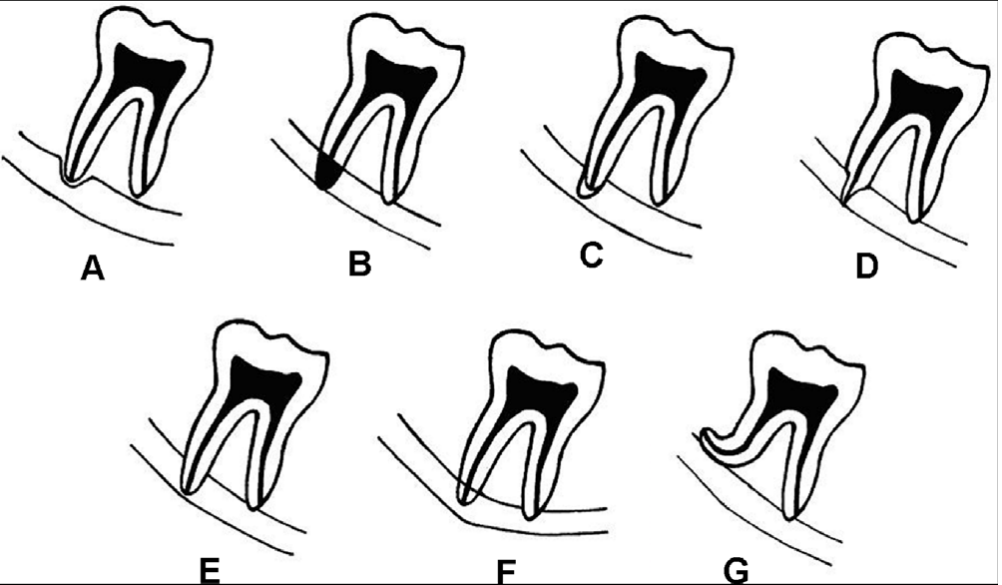
**Radiological signs; (A) narrowing of canal, (B) darkening of root, (C) dark and bifid apex of root, (D) narrowing of root, (E) interruption of white line of canal, (F) diversion of canal, (G) deflection of root**.

**Figure 3:**
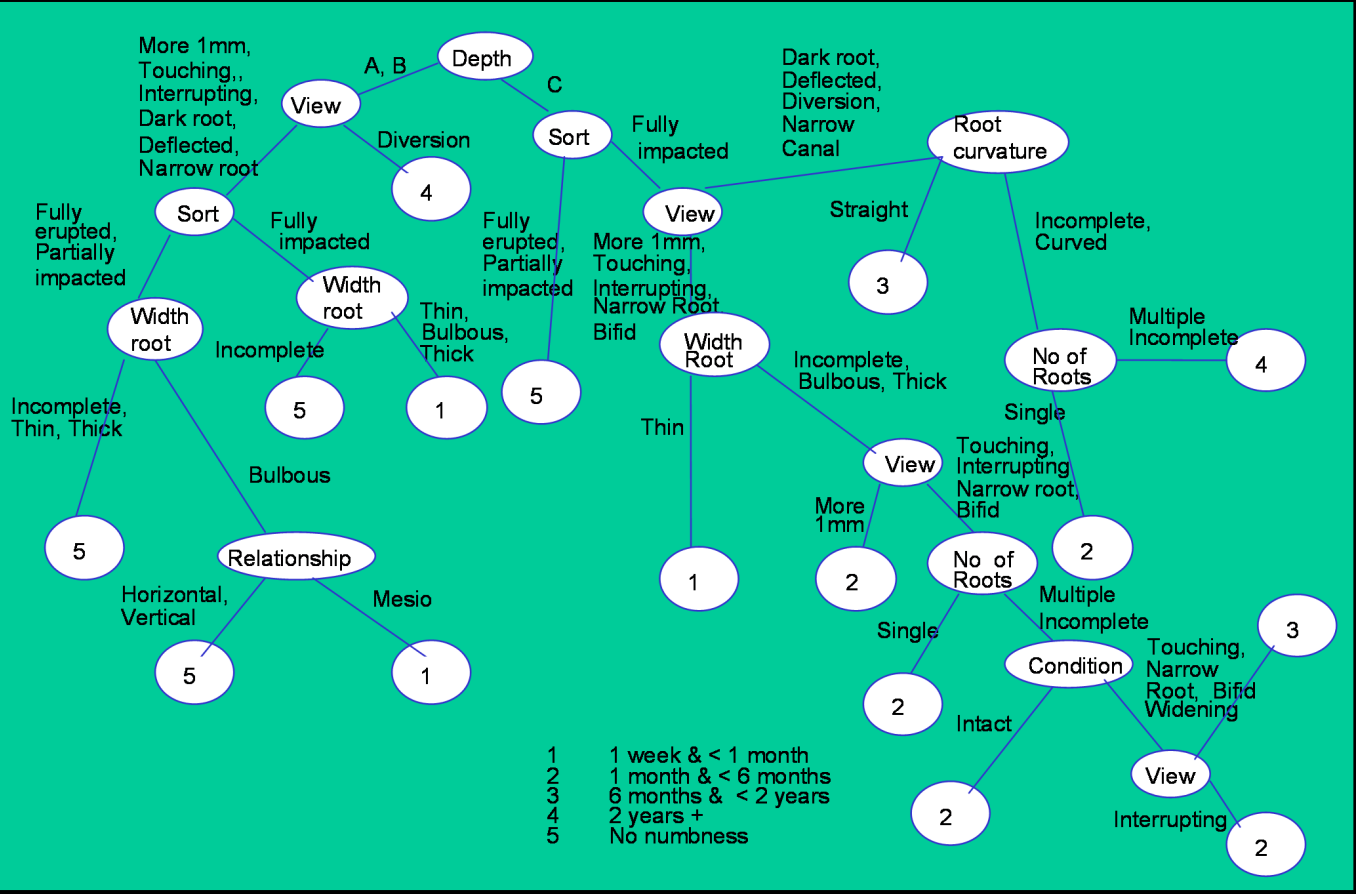
**Tree split with Pearson x^2 ^Example 1: if the depth of impaction is A or B and the view of the x-ray: more than 1 mm, touching, interrupting, dark root, deflected or narrow root and the type fully erupted or partially impacted and the width of the root incomplete, thin or thick then there will be no predicted numbness**. Example 2: if the depth of the impaction is level C and the type fully impacted and the view more than 1 mm, or touching or interrupting or narrow root or bifid apex and the width of the root incomplete, bulbous or thick and the view touching, or interrupting or narrow root, or bifid foot and the number of roots multiple or incomplete and the condition of the gum widening and the view touching, narrow root or bifid foot, predicted numbness will be between 6 months and less than two years. Example 3: if the depth of the impaction was level A or B and the view diversion, there will be numbness for more than two years.

The first study [1] (prediction of complications using OPGs) enabled us to accurately assess the relative risk of temporary and permanent inferior alveolar nerve paraesthesia postoperatively. By examining the seven identified radiological signs (Figure 2) and applying them in our classification tree (Figure 3), a decision can be formulated whether the operation should be undertaken by a junior or senior surgeon or whether local, general anaesthesia or intravenous sedation should be employed.

**Figure 4:**
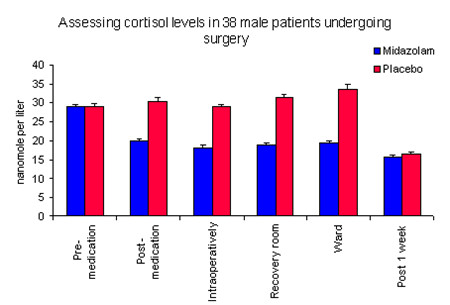
**Nerve injury in relation to recovery time; also a proposed plan for follow-up, exploration and surgical intervention**.

It was concluded from the second study [2] (assessment of stress and anxiety) that small doses of midazolam may have a significant beneficial effect on the patient pre-, intra- and postoperative stress and anxiety by significantly reducing the level of cortisol in saliva. Cortisol level was simply used as an indicator of perceived and physiological stress (Figure 4). Anxious patients can be prescribed benzodiazepines prior to surgery which can reduce the perceived stress.

**Figure 5:**
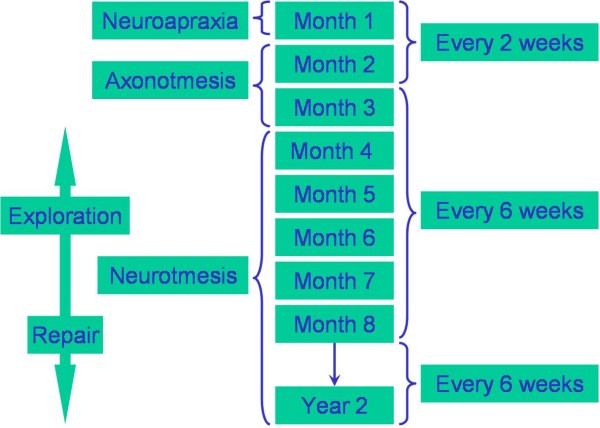
**Standard collection for all patients (6 salivary samples) to assess the cortisol level in saliva (reflects the plasma cortisol level)**.

The higher rate of postoperative complications in the group treated by junior surgeons suggests that at least some of the complications might be related to surgical experience [3,4]. Nerve complications can vary and have different aetiologies; one relatively consistent feature is their effect on the recovery and intervention time (Figure 5). Long-term complications (i.e. neurological deficit) appear higher in the hands of junior surgeons (Table 1). This highlights potential training issues: academic teaching, clinical teaching, clinical supervision, amount of cases performed by a junior surgeon and surgical competencies. It also places emphasis on ethical issues such as the patient's right to know who is performing the procedure and that person's seniority, experience and complication rate.

**Table 1:** Incidence of each type of postoperative complications in relation to seniority of the surgeon undertaking the procedure.

**Complication**	**Resident n (%)**	**Specialist n (%)**	**P value**
Trismus	74 (14.3)	49 (8.6)	0.003
Swelling	62 (12.0)	63 (11.1)	0.643
Bleeding	15 (2.9)	33 (5.8)	0.020
Sore throat	9 (1.7)	4 (0.7)	0.117
Dry socket	99 (19.1)	39 (6.9)	<0.001
Delayed healing	14 (2.7)	8 (1.4)	0.129
Abscess	7 (1.4)	7 (1.2)	0.860
Infection	54 (10.4)	25 (4.4)	<0.001
			
Lip numbness 2 weeks	15 (2.9)	4 (0.7)	0.012
Tongue numbness 2 weeks	24 (4.6)	6 (1.1)	<0.001
Lip numbness 2 years	7 (1.4)	1 (0.2)	0.056
Tongue numbness 2 years	9 (1.7)	2 (0.4)	0.048
			
Any complication	330 (63.7)	223 (39.2)	<0.001

## Impact on the patients, professionals and future healthcare service

### Prediction of surgical difficulty and inferior alveolar nerve injury [1]

One important aspect of the first study on assessment of the degree of difficulty stems from it allowing the necessary information to be presented to the patient about the risks of the procedure. Much of the previously available information about complications was either old data or has been accumulated from a series of retrospective studies. Although all of the information in this study was collected retrospectively, the data all derived from one centre using surgeons with a variety of skill mixes.

More importantly, the derivation and use of the classification tree could aid in the case selection. For example a wisdom tooth with a certain classification tree rating might be suitable for an undergraduate. After a suitable period of satisfactory skill assessment at this level, a junior dental surgeon would be allowed to move on to higher rated teeth and so on throughout the training continuum, so that the really difficult cases would only be performed by specialist. At the present time this work is being validated - that is to say, a new group of patients are having their radiographs scored and these scores compared with outcomes in terms of complications. Again, this approach is of great interest in training as a series of prognostic indicators can be identified and scored. In this way, safe training progression can be developed with appropriate skills assessment to ensure competence. This would allow struggling trainees to have more time to hone their skills, but also allow gifted surgeons to fast-track through training.

### Assessment of patient's stress and anxiety [2]

This study clearly demonstrated a previously unmet need in day case surgery. There has been a trend to use no pre-medication in the day care setting. This study clearly confirmed that stress and anxiety levels in preoperative patients are high and indicates the value of using short acting anxiolytics in reducing stress and anxiety without delaying recovery or creating other untoward effects. This is powerful information for patient groups who often feel surgeons and anaesthetists hide behind safety issues when withholding pre-medication from patients undergoing day care surgery. This work has also stimulated interest from a number of pharmaceutical companies to consider other novel drug delivery systems for day case use. One such technique that is being investigated is the use of inhaled pre-medication. This has several advantages in that the drug is not given orally (safer in a fasted pre-operative patient) and the onset of anxiolysis is much faster.

This study has identified a problem, also suggested a solution that is available now (sublingual midazolam) and has stimulated future research. It is important to note that the original study was performed on adults. If a rapidly acting effective preparation were made available for children, then this could be deployed whenever required.

Of more relevance however is the applicability of these findings in other similar surgical situations. The conclusions from the study are likely to be valid for any invasive procedure. Dental treatment (root fillings, surgical extractions, complex bridgework and implants), endoscopic procedures (gastroscopy and colonoscopy), lumbar puncture...etc. Some of these investigations are currently performed with intravenous sedation, but there is the potential advantage of inhaler based administration and other such alternative routes giving good sedation but for a shorter duration, making the patients recovery and return to a normal existence easier (for example enabling them to adopt normal travelling arrangements).

### Assessment of postoperative complications and the surgeon's experience [3,4]

This study opens the debate about impact of the skill level of the surgeon on postoperative complications. Interestingly, the outcomes appeared to be independent of the level of supervision of the surgeon. This has huge implications for patients. If the general public were aware of the differences in complications between specialists and generalists, they would demand their operations be carried out only by experienced, fully trained staff. We expect these comparative figures to look much worse when undergraduates are compared with specialists. Again, the maxim, "no students, no dentists" applies, so it is important to find some mechanism to allow the safe surgical progression from simple extraction to complex surgical removal of wisdom teeth.

### Psychosocial impact

Much of the third molar surgery literature focuses on refining clinical assessment and management in order to achieve improved outcomes for the patient and surgeon. Regardless of these factors, there will probably always be some third molar surgery complications that could be classified as chronic or long-term. However, there is currently an inadequate body of literature focusing on the psychosocial issues that can affect the quality of life of patients with such long-term complications. These include factors such as understanding the emotional impact of pain and any long-term sensory deficit; devising mechanisms to develop appropriate social support coping mechanisms and responses to stress whilst maintaining treatment adherence. Healthcare professionals are becoming increasingly aware of the impact psychosocial issues may have on wound healing in general. Therefore, there is a need to consider alternative intervention measures for patients for whom healing alone may not be a realistic option.

It is also wise to remember that some patients are more anxiety prone and their cortisol levels are likely to be high in a normal situation. Exposing them to hospital environment might actually lead to severe anxiety or even panic attacks. This group of patients is more likely to develop postoperative complications and suffer poor wound healing, and hence have a troublesome journey. Fortunately, no patient in our study experienced such extreme events. If such patients were identified in the assessment phase, full psychological counseling would be advised.

The stress and anxiety of the surgeon with limited experience may also negatively affect the patient directly and might be related to a number of postoperative complications and delayed or poor wound healing, and hence relatively poor prognosis. Other factors that have been implicated include the practitioner's approach to the patient. Anxiety and stress can be significant when training of an undergraduate or junior surgeon takes place in a clinic where the patient retains consciousness and can hear the conversation. Several studies have showed that psychological interventions in such circumstances can elevate the patient's pain threshold and be associated with a lower complication rate [5].

### Impact on the profession, education and research

All university dental schools provide undergraduate training in oral surgery in accordance with the guidelines provided by the General Dental Council (GDC). However, the courses vary with regard to the departments involved and the level of student participation. Training is likely to comprise basic familiarisation with the principles of oral surgery and the factors that determine which patients should be referred for consultant advice.

Non-surgical removal of third molars is usually practiced at the undergraduate level; however, some dental schools have introduced a new scheme which allows dental undergraduates to perform surgical extractions. Irreversible anatomical damage has been reported and re-treatment may be complex, or impractical. Usually these procedures take place under senior supervision or the senior acting as an "assistant". However, compensation arising from litigation can affect both the cost and conditions of professional indemnity.

A number of issues underlie the practice of oral surgery, including the preparation and continuing surgical education of practitioners. In past times, the doctor was generally not troubled by litigation from patients presenting as emergency cases (i.e. severe jaw pain), but this aspect of practice has changed. Litigation by patients now significantly affects dental and surgical practice and vicarious liability often affects hospitals.

It is expected that the development of national uniformity in research, teaching and assessment of clinical skills will lead to some progress in developing a high-standard service across the country and cut down the number of litigations. Hospitals need to adopt a risk management approach, including assessment of the competence of dental practitioners, particularly in procedural skills.

### Lessons learnt

While it is predictable that patients should have complications from surgery that depend on the experience of the surgeon, the differential found between specialists and trainees was surprising and unacceptable. We realised that it should now be possible to predict the degree of difficulty and complication rate in third molar surgery. This knowledge should inform the process of patient selection for undergraduate and postgraduate training so that the public is reasonably protected. Of course, this work needs validation and the next logical step is to see if preoperative assessment and the application of the classification decision making tree can be used predictively. When this validation has been completed, it is our intention to set indicative standards for different levels of trainees with progression to more complex procedures following skills assessment. In addition, the role of anxiolytics in third molar surgery has been substantiated. Patients can choose to have or can be at least offered these medications when they report a history of fear and anxiety in the assessment stage.

What is more, if this approach proves to be successful, the process could be applied in a number of areas in surgical training so that experience can be optimised without exposing the general population to unnecessary risks.

The evolution of the concepts related to the subject has lead from one question to another until in reflection not only a substantial body of work, but also something truly worthwhile had been created of benefit on many levels; from clinical training to patient comfort and safety.

## Conclusion

The results of those three studies have revolutionized thinking when it comes to management of patients requiring removal of third molars. It is expected that this will have an impact on the patients, professionals and future healthcare service, potentially leading to revised clinical guidelines and the implementation of special protocols in dental hospitals. Psychosocial impact and the impact on the profession, education and future research should not be ignored.

## Competing interests

The authors declare that they have no competing interests.

## Authors' contributions

WJ, TU, PK, SA, JR, EM, PM, CH: contributed to conception and design, carried out the literature research, manuscript preparation and manuscript review.

All authors read and approved the final manuscript.
